# Pet cat personality linked to owner‐reported predation frequency

**DOI:** 10.1002/ece3.9651

**Published:** 2023-01-24

**Authors:** Marion Cordonnier, Amira Perrot, Nicolas Ferry, Elsa Bonnaud, Emmanuelle Baudry

**Affiliations:** ^1^ CNRS, AgroParisTech, Ecologie Systématique Evolution, Université Paris‐Saclay Orsay France; ^2^ Lehrstuhl für Zoologie/Evolutionsbiologie, University of Regensburg Regensburg Germany; ^3^ Bavarian Forest National Park Grafenau Germany

**Keywords:** depredation, domestic cats, personality traits, urbanization

## Abstract

The domestic cat, *Felis catus*, is one of the most popular and widespread domestic animals. Because domestic cats can reach high population densities and retain at least some tendency to hunt, their overall impact on wildlife can be severe. Domestic cats have highly variable predation rates depending on the availability of prey in their environment, their owners' practices, and individual cat characteristics. Among these characteristics, cat personality has recently been hypothesized to be an important factor contributing to variations in the hunting activity of cats. In this study, we surveyed 2508 cat owners living in France about their cats' personalities, using the Feline Five personality framework, and the frequency with which cats bring home prey. Personality traits were analyzed using factor analysis and related to predation frequency using cumulative logit models. For both birds and small mammals, cats with high levels of extraversion or low levels of neuroticism had significantly higher frequencies of prey return. Owners whose cats had low levels of agreeableness or high levels of dominance reported a significantly lower frequency of bird return. Personality differences therefore seem to contribute to the high variability in predation rates among domestic cats. We also found that the owner‐reported prey return frequencies were significantly higher for cats spending more time outdoors, for non‐pedigree cats, and for owners living in rural or suburban areas as opposed to urban areas. By contrast, we did not detect an effect of cat sex or age on their reported prey return rates.

## Graphical abstract

Because domestic cats can reach high population densities and generally retain at least some tendency to hunt, their overall impact on wildlife can be severe, leading to increasing international interest and concern. Domestic cats have highly variable predation rates, depending in part on individual cat characteristics, but the reasons for this high level of variation are poorly understood. In our study, we used surveys of more than 2500 cat owners living in France to show that personality differences contribute strongly to the large variability in predation rates among domestic cats.

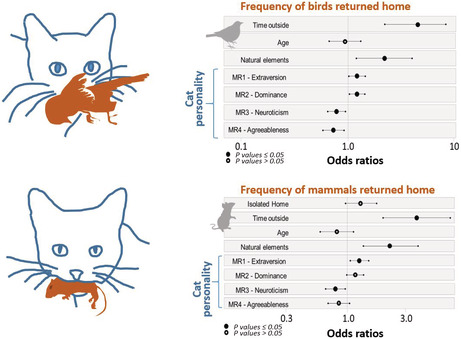

## 1 INTRODUCTION

The domestic cat, *Felis catus*, is currently one of the most common carnivores in the world (O'Brien & Johnson, 2007). Cats are generalist predators introduced by humans globally, and their potential impact on wildlife is the subject of growing international interest and concern (Crowley et al., 2020a; Loss & Marra, 2017). They hunt many types of prey, including invertebrates and vertebrates, mainly mammals, birds, and reptiles (e.g., Barratt, 1997; Castañeda et al., 2019, 2020). The ecological impacts of cats have been shown to be particularly severe on island ecosystems, where island vertebrates have never coexisted with such introduced mammalian carnivores, and cats are a major driver of extinctions of insular endemic birds, mammals, and reptiles (Bonnaud et al., 2012; Doherty et al., 2016; Medina et al., 2011; Palmas et al., 2017). On continents, cats have been estimated to be responsible for high vertebrate mortality (e.g., Blancher, 2013; Loss et al., 2013; Murphy et al., 2019), although the extent to which their predation represents a form of compensatory or additive mortality is currently under debate (Loss & Marra, 2017), as they consume the most abundant prey and rarely the most vulnerable or declining species.

The majority of research to date has focused on the behavior and impacts of feral cats (see the review of Loss et al., 2022), which are dependent on the abundance and availability of natural prey species. However, most pet cats that are fed by their owners retain some tendency to hunt (Thomas et al., 2012; Tschanz et al., 2011), and as they can reach very high population densities in areas where humans are also numerous (Baker et al., 2005; Sims et al., 2008), their overall impact can be severe. Several studies have shown that pet cats have highly variable predation rates (Loyd et al., 2013; Tschanz et al., 2011). For example, Baker et al. (2008) and Thomas et al. (2012), respectively, showed in the cities of Bristol and Reading, UK, that approximately 60% of pet cats did not return prey home in the study period, highlighting the importance of identifying the factors that determine the predation rates of individual cats. Cecchetti, Crowley, Goodwin, and McDonald (2021) recently reviewed the drivers of hunting behavior in domestic cats. For the authors, whereas general cat hunting is mainly driven by evolutionary constraints and the associated physiological and nutritional requirements, the causes of variation in hunting behaviors among pet cats mainly relate to prey availability in the environment and the owners' practices. These practices include the level of outdoor access given to their cats, the amount and quality of the food provided, and the amount of time spent playing with the cat (Cecchetti, Crowley, & McDonald, 2021).

Variations in hunting activity have also been linked to the individual characteristics of cats such as their sex, age, and body size (Kays & DeWan, 2004; Moseby et al., 2015), although a number of studies have failed to find an association with these factors (Cordonnier et al., 2022; Loyd et al., 2013; Tschanz et al., 2011; Woods et al., 2003). Recently, Cecchetti et al. (2021a) hypothesized that personality could be a significant factor contributing to variations in hunting activity between cats. Over the past few decades, it has been recognized that in numerous animal taxa ranging from invertebrates to vertebrates, individuals show different behavioral tendencies that are consistent over time and across ecological contexts, a phenomenon commonly known as animal personalities (Réale et al., 2007, 2010; Wolf & Weissing, 2012). For example, boldness, aggressiveness, or sociability are commonly studied animal personality traits (Réale et al., 2007). Personality traits are frequently correlated: for example, animals that are bolder in risky situations also have a tendency to be more aggressive toward conspecifics, resulting in what is known as “behavioral syndromes” (Sih et al., 2004). Animal personalities have substantial consequences for numerous ecological processes (Brehm et al., 2019; Spiegel et al., 2017; Wolf & Weissing, 2012). Regarding predator–prey interactions, several studies have shown that individual differences in predator behavior can influence hunting (Pettorelli et al., 2011). For example, in several predator fish species, bolder individuals have a markedly higher predation rate compared with shyer ones (Ioannou et al., 2008; Rhoades et al., 2019). In their review of the drivers of hunting behavior in domestic cats, Cecchetti et al. (2021a) speculated that cats with certain personality traits, particularly those with high levels of boldness and extraversion, could potentially be more motivated to hunt wild prey. To our knowledge, this hypothesis has never been investigated.

Currently, the assessment of personality traits in domestic cats is most often based on surveys of people familiar with the animals, usually their owners (Bradshaw, 2016; Wedl et al., 2011), as this is both a reliable and time‐efficient method (Bennett et al., 2017). These studies (reviewed in Gartner & Weiss, 2013; Vitale Shreve & Udell, 2015; Mikkola et al., 2021) usually produced between one and seven personality factors, with the three most common factors being the personality traits of sociable, dominant, and curious, albeit with varying names. In this study, we used the Feline Five personality model of Litchfield et al. (2017), which consists of five personality dimensions in domestic cats: neuroticism, extraversion, dominance, impulsiveness, and agreeableness (see further details in the ‘Section 2’).

In this study, our primary objective is to determine whether the personality traits of pet cats are related to their hunting activity. To this end, we surveyed a large sample of cat owners living in France and estimated the personality traits of their cats using the Feline Five personality model of Litchfield et al. (2017) as well as the frequency of birds and mammals returned home by the cats as reported by their owners. We expected that cats with “low neuroticism (boldness, leading to travelling, exploring) or high extraversion (curiosity, leading to boredom), would potentially be more interested in hunting wild prey” (Cecchetti, Crowley, & McDonald, 2021), To control for potential confounding factors, we also included questions about variables previously shown to influence pet cat predation: type of environment around the home, time spent outdoors, individual characteristics, and breed (Castañeda et al., 2019, 2020; Cordonnier et al., 2022; Kauhala et al., 2015; Lepczyk et al., 2004; Robertson, 1998; Salonen et al., 2019).

## 2 MATERIAL AND METHODS

### 2.1 Questionnaire design and dissemination

A questionnaire was developed to collect information on French pet cats regarding their personality (five personality traits model) and the frequency with which their owners observed them bringing home birds and small mammals (ranging from never to very often, defined as once a week or more). To control for potential confounding factors, additional information was gathered on other characteristics of the cats (sex, age, and breed) and their living conditions (type of dwelling, type of the area around the dwelling, amount of time spent outdoors). The questionnaire was hosted online on the Google Form platform.

Households in France with at least one pet cat were targeted through postings on social media. We asked respondents with multiple cats to focus on one particular cat, the one they wanted. The survey was anonymous, and no personal information was collected from the respondents. In the introduction part of the survey, respondents indicated their consent to participate in the study. The study complied with the legal requirements in France: as no personal information was collected, ethics approval was not mandatory, as was confirmed by the Research Ethics Committee of Paris‐Saclay (Polethis, report from January 4, 2021).

The questionnaire consisted of four sections (Appendix S1). The first section focused on the cat characteristics: sex (female, male, unknown), age (<1 years, 1–2 years, 2–10 years, over 10 years, unknown), breed (Bengal, Birman, British Shorthair, Chartreux, Maine Coon, Persian, Ragdoll, Savannah, Sphynx, Siamese, Turkish Angora, non‐pedigree, European, other, unknown). Note that in France “European” means “non‐pedigree.” We offered the two options, because some owners might have been more familiar with one word than the other. The second section focused on the living conditions of the cat: type of housing (apartment without balcony, apartment with balcony, subdivision house, individual house), type of environment (urban, suburban, or rural area), time spent by the cat outdoors daily (none, limited [<1 h], moderate [1–5 h], long [>5 h], all the time [just comes back to eat]), daily time spent by the owner with the cat (none, limited [<1 h], moderate [1–5 h], long [>5 h]).

The third section involved assessing the personality traits. Litchfield et al. (2017) determined that the personality profiles of cats are organized around five factors that represent traits related to neuroticism, extraversion, agreeableness, dominance, and impulsivity. Each factor can be evaluated using a list of adjectives that have varying correlations with the trait in question. In our questionnaire, to ensure short response times and thus high completion rates (e.g., Plowman et al., 2013), we selected 15 of the 52 adjectives used in the original study of Litchfield et al. (2017). For each of the five personality traits, we selected three adjectives based on two criteria. First, we selected adjectives with unequivocal translations in French to avoid ambiguity for the respondents. Second, we used the factor scores of Litchfield et al. (2017) for each adjective to select those with a high correlation with the relevant personality trait and a low correlation with the four others in order to facilitate the interpretation of the results. For example, the adjective affectionate was selected, because it is readily translatable in French and has a high correlation with the personality trait of agreeableness and a low correlation with the four other personality traits. In English, we chose the following 15 adjectives. For neuroticism we chose: shy, calm (negative loading), and fearful of other cats; for extraversion: smart, vigilant, and persevering; for agreeableness: affectionate, friendly to people, and solitary (negative loading); for dominance: bullying, dominant, and aggressive to other cats; and for impulsiveness: impulsive, predictable (negative loading), and distractible. Each of these 15 adjectives was presented to the owners who could choose between strongly disagree, disagree, neither agree nor disagree, agree, and strongly agree.

The final section of the questionnaire focused on the prey returned home as observed by the owners and included the reported frequency of return of birds and small mammals (daily, 1–6 times per week, 2–3 times per month, 1–3 times per trimester, 1–3 times per year, never). At the end of the survey, we added an optional open‐ended question to give the owners the opportunity to add any comments that they wished to share about their cat and to communicate any insights that may have been overlooked in the survey (Harland & Holey, 2011).

The social network Facebook®was chosen as a channel to disseminate the questionnaire. This network has a large number of user groups dedicated to cats, which made it possible to conduct a large‐scale study. The questionnaire was distributed in 23 French‐speaking groups from February 9, 2021, to March 14, 2021, which allowed us to collect a total of 3217 responses. The dataset was deposited in the Mendeley repository (https://data.mendeley.com/datasets/ht5p5pg7b7/1; DOI: 10.17632/ht5p5pg7b7.1).

### 2.2 Data treatment and statistical analyses

All analyses were conducted using R v.4.2 (http://www.R‐project.org). For all statistical tests, the level of significance was set at *p* < .05.

#### 2.2.1 Factor analysis of personality structure

To ensure that the personality traits of the cats were reliably described by the owners, we removed from the analyses the surveys indicating that the owners did not spend any time with their cat (*n* = 10) and those with comments that prevented their use to study the personality traits (e.g., respondent not the cat owner, recently adopted cat, cat with a major health issue; *n* = 21). Like all animal species, cats go through different stages of development, and in juveniles, personality and predatory behavior are not yet stable (Lowe & Bradshaw, 2001). Following Litchfield et al. (2017), we conservatively excluded cats aged under 1 year (*n* = 614) from the dataset. Finally, the responses with missing data in the set of personality adjectives were also removed (*n* = 64). The final dataset included 2508 responses.

We performed exploratory factor analysis on the personality variables (15 adjectives evaluated by the owners) to first determine the number of personality traits to be extracted and then estimate the values of each trait for each cat (Hutcheson & Sofroniou, 1999). We initially ensured that the data were suitable for factor analysis using Bartlett's test of sphericity, which was significant (*p* < .01), and the Kaiser‐Meyer‐Olkin (KMO) criterion, which had an overall value of 0.722 (depending on the authors of the statistical tests, a value above 0.5 or 0.6 is considered to mean that the sampling is adequate). Both indicators thus show that the data were suitable for factor analysis (Hutcheson & Sofroniou, 1999; Kaiser, 1974). To determine the number of factors – here, personality traits – to extract, we used the empirical Kaiser criterion (see Braeken & Van Assen, 2017 for details) and parallel analysis with principal component analysis (PCA) (comparing the eigenvalues obtained to those generated from a Monte‐Carlo simulated matrix), which indicated that four factors should be retained. We therefore choose to retain four factors, and used maximum likelihood factor analysis to extract them from the 15 adjectives. We obliquely rotated these factors (i.e., correlations between them were allowed), because personality traits are frequently correlated, as shown by the existence of personality syndromes. As in previous studies (e.g., Weiss et al., 2015), to interpret the factors, we defined salient loadings as those equal to or greater than |.4|. The four factors that we obtained correspond approximately a posteriori to Litchfield et al. (2017) extraversion (MR1), dominance (MR2), neuroticism (MR3), and agreeableness (MR4) (see ‘Section 3’ below).

#### 2.2.2 Breed and personality

We tested the link between breed and personality traits by computing the Euclidian distance between personality profiles for each pair of cats to produce a resemblance matrix from which we conducted a nonparametric (permutational) analysis of variance (permanova; package vegan; Oksanen et al., 2007) using 999 permutations to test whether personality profiles differed according to breed. We then performed discriminant analysis using a non‐parametric version of Pillai's test to evaluate the significance of the eigenvalues (package ade4; Dray & Dufour, 2007). To ensure to have enough statistical power, we removed five breeds with <60 individuals (Chartreux, *n* = 28; Savanah, *n* = 20; Sphinx, *n* = 57, Siamese, *n* = 49, Turkish Angora, *n* = 21), leading to a dataset of 2162 responses. We regrouped the “Non‐pedigree” and “European” cats under the “Non‐pedigree” label, as both terms are used to describe the same type of cat in France (the European Short Hair breed exists but is extremely rare in France).

#### 2.2.3 Factors influencing owner‐reported frequency of prey brought home

To run the subsequent analyses regarding predation, additional responses were excluded from the previously described dataset: cats living in apartments with minimal outdoor access as well as cats living in houses but without daily outdoor access (*n* = 1217), owners of four or more cats (Cordonnier et al., 2022) who would supposedly have difficulty determining which cat brought home which prey (*n* = 188), incomplete responses (*n* = 2), and cats belonging to the Bengal breed (*n* = 36) because when the survey was posted on a Bengal cat Facebook group, several people suggested in the comments that participants give false answers to questions relating to predation. The final dataset included 719 responses. Since the response variables for reported predation frequencies were ordered (0: never, 1: 1–5 times a year, 2: 5–10 times a year, 3: 1–3 times a month; 4: once a week or more), two cumulative logit models (CLMs) were adjusted (McCullagh, 1980), with the reported frequencies of predation taken as response variables: CLM1: birds, CLM2: mammals. Each model incorporated 11 predictor variables: cat sex (0: male, 1: female), cat age (ordinal variable: 0: 1–2 years, 1: 2–10 years, 2: 10 years and older), cat breed (Birman, British Shorthair, Maine coon, Persian, Ragdoll, non‐pedigree), type of housing (0: in a subdividion, 1: detached home), abundance of natural elements (tree, bushes, grass, etc.) around the place of residence (0: low, 1: moderate, 2: high), urban level (rural, suburban, urban), time spent daily outdoors (0: limited, 1: moderate, 2: long, 3: all the time), and the four quantitative personality traits (MR1 to MR4). To avoid potential multicollinearity issues, we ensured that all Variance Inflated Factors (VIFs) were under the threshold of 2 (O'Brien, 2007). The two models were fitted using the package ordinal (Christensen, 2015). For both models, we used a stepwise selection by sequential replacement to identify the subset of variables in the dataset resulting in the best performing model with the lowest prediction error (Hegyi & Laczi, 2015; Venables & Ripley, 2013). Wald tests were performed on the predictor variables. The quality of the model estimates was monitored using Pearson residuals (package sure; Greenwell et al., 2018). For the qualitative variables, post hoc tests (including a Holm correction) were performed using a self‐designed contrast matrix (package lsmeans; Lenth & Hervé, 2015).

## 3 RESULTS

### 3.1 Factor analysis of personality structure

**FIGURE 1:**
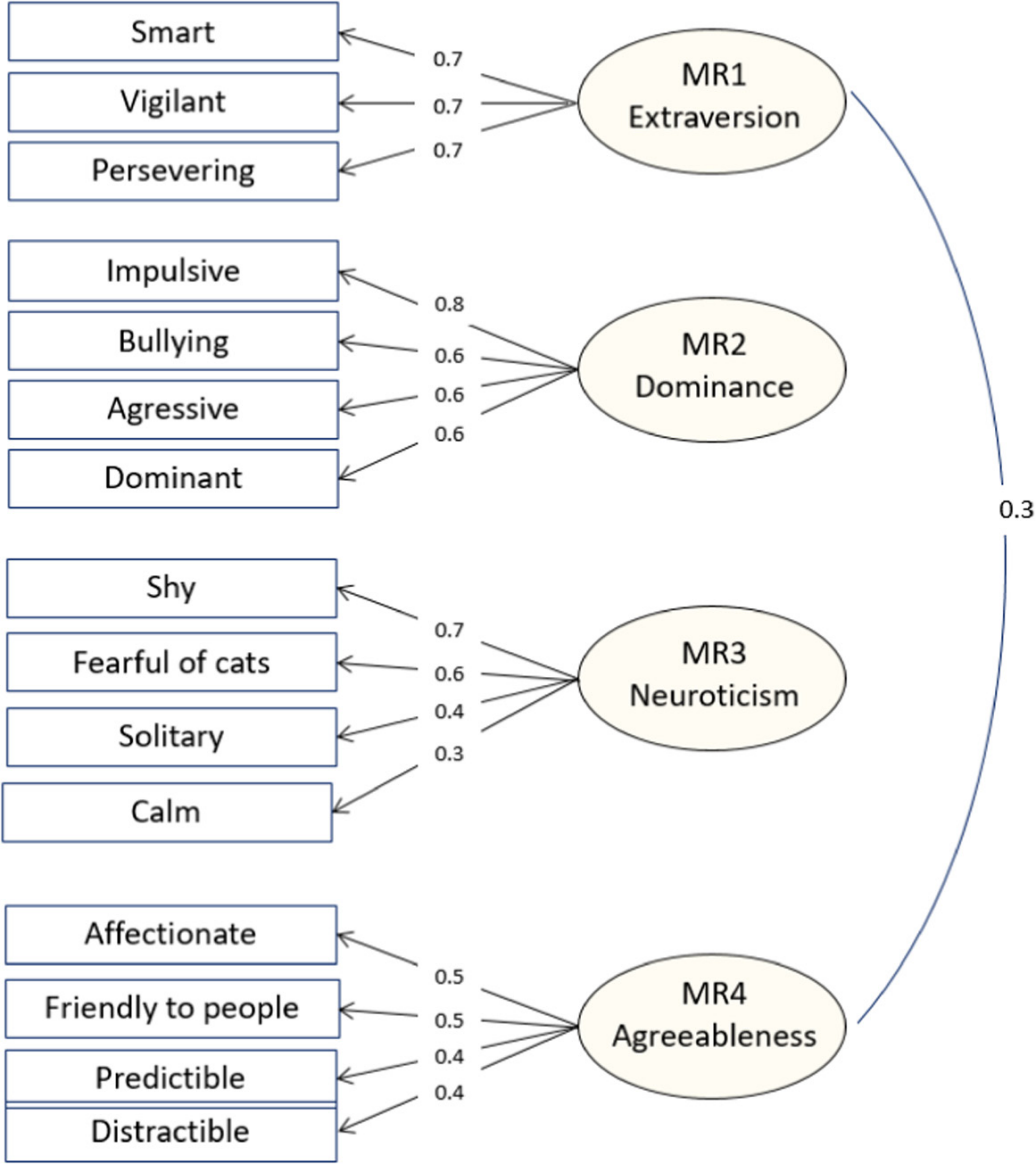
Exploratory factor analysis model of cat personality with four factors.

We performed exploratory factor analysis on the 15 cat personality items selected from the list of Litchfield et al. (2017). We obtained four factors which explained 30.9%, 28.6%, 23.8%, and 16.7% of the variance, respectively (Table 1a). The value of the correlations between them was relatively small, ranging from −0.01 to 0.30 (Table 1b). Table 1a and Figure 1 show the loadings of these four extracted factors for the 15 cat personality items. As he four personality dimensions were very similar to those extracted by Litchfield et al. (2017), we kept the same names: extraversion, dominance, neuroticism, and agreeableness. However, we did not find their fifth factor, impulsiveness, which was expected to be associated with the adjectives impulsive, predictable, and distractible. In our dataset, the impulsive adjective was strongly loaded on the Dominance factor, whereas predictable and distractible were moderately loaded on the Agreeableness factor (Table 1a).

**TABLE 1a:** A‐four extracted personality factors and their loadings for the 15 cat personality items. Salient factor loadings (above |0.40|) are shown in bold. h2 measures communalities, the shared variance with the other items, whereas u2 measures uniqueness, the variance not explained by the other items

	Dominance (MR1)	Extraversion (MR2)	Neuroticism (MR3)	Agreeableness (MR4)	h2	u2
Shy	−0.05	0.02	**0.73**	−0.07	0.55	0.45
Calm	−0.32	0.13	0.35	0.24	0.31	0.69
Fearful of cats	0.05	−0.03	**0.56**	0.16	0.31	0.69
Smart	−0.03	**0.73**	−0.06	0.10	0.59	0.41
Vigilant	0.01	**0.74**	0.20	−0.08	0.58	0.42
Persevering	0.12	**0.67**	−0.15	0.02	0.49	0.51
Affectionate	−0.11	0.33	−0.03	**0.51**	0.49	0.51
Friendly to people	−0.05	0.06	−0.39	**0.48**	0.46	0.54
Solitary	0.17	0.12	**0.41**	−0.16	0.26	0.74
Bullying	**0.61**	−0.01	−0.09	−0.08	0.41	0.59
Dominant	**0.63**	0.13	−0.12	−0.06	0.44	0.56
Aggressive	**0.60**	0.02	0.23	0.03	0.41	0.59
Impulsive	**0.75**	−0.01	0.02	0.08	0.55	0.45
Predictable	0.10	0.02	0.13	**0.43**	0.19	0.81
Distractible	0.22	−0.15	0.08	**0.42**	0.18	0.82
Prop. Var	0.13	0.12	0.10	0.07		
Prop Var explained	0.31	0.29	0.24	0.17		

**TABLE 1b:** Correlations between the four extracted personality factors

	Dominance (MR1)	Extraversion (MR2)	Neuroticism (MR3)	Agreeableness (MR4)
Dominance (MR1)	1.00			
Extraversion (MR2)	0.07	1.00		
Neuroticism (MR3)	−0.01	0.06	1.00	
Agreeableness (MR4)	−0.14	0.30	−0.16	1.00

### 3.2 Breed and personality

We confirmed that personality is influenced by breed (permanova; *F*_6_ = 14.926, *p* = .001). Discriminant analysis was significant (non‐parametric version of Pillai's test; Obs. = 4.7175; Exp. = 0.0028; *p* = .001). The analysis separated mainly non‐pedigree and Bengal cats from the other breeds along the first axis, suggesting higher dominance (MR2), higher extraversion (MR1), and lower agreeableness (MR4) in these two groups. The second axis separated mainly Bengal and Main Coon cats from the other breeds, suggesting lower neuroticism in these two breeds compared with other cats (Table 2, Figure S1).

**TABLE 2:** Discriminant analysis of the relationship between breed and personality

	Discriminant axis 1	Discriminant axis 2
MR2	−0.64	−0.11
MR1	−0.39	−0.65
MR3	−0.30	0.98
MR4	0.51	0.34

	Discriminant axis 1	Discriminant axis 2
EUR	−0.19	0.10
BEN	−0.19	−0.69
BRI	0.42	−0.04
MCO	0.21	−0.39
PER	0.34	0.18
RAG	0.65	0.18
SBI	0.29	−0.15

### 3.3 Factors influencing owner‐reported frequency of prey brought home

**FIGURE 2:**
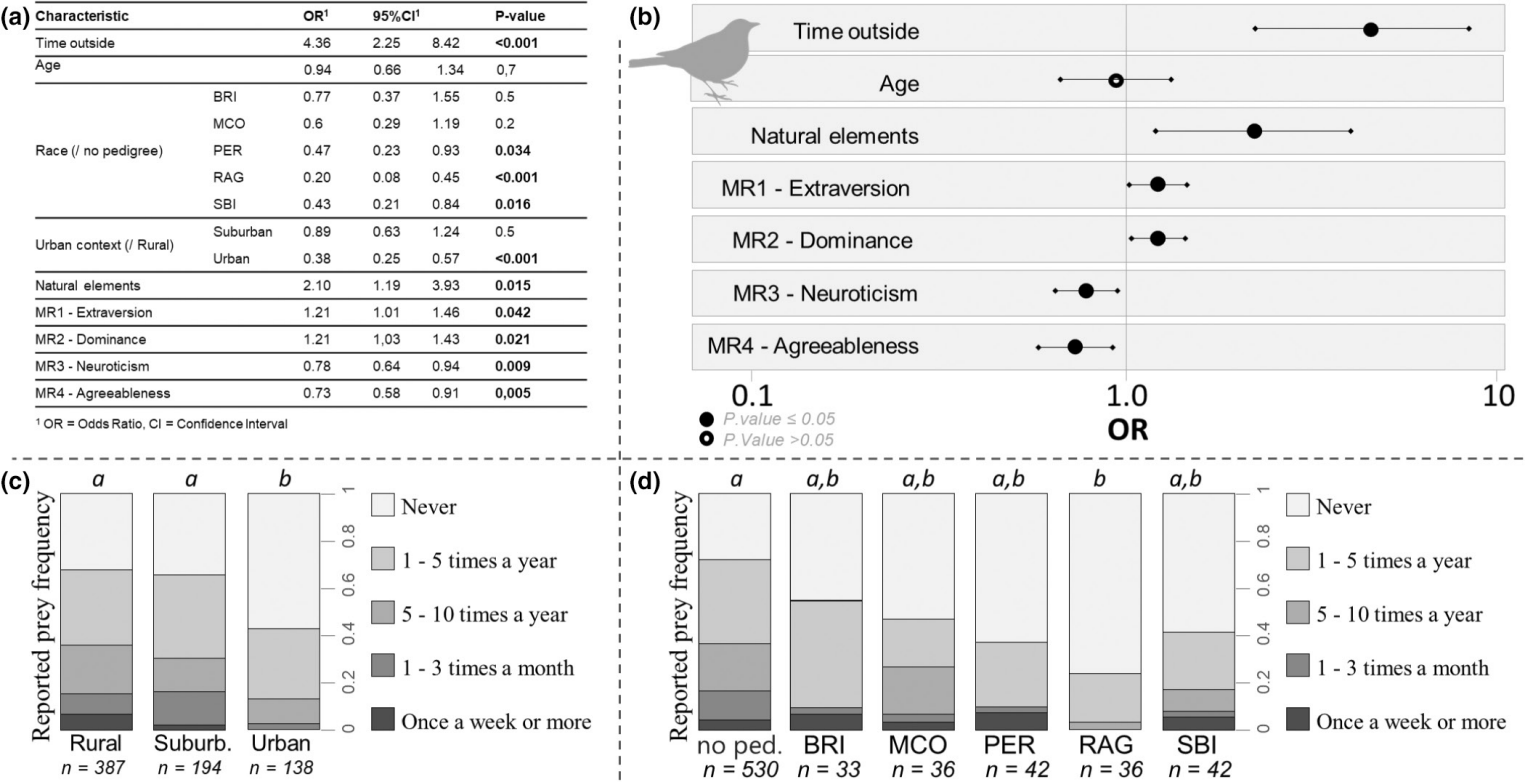
Results of the final CLM1 model of the owner‐reported frequencies of birds returned home (*n* = 719). (a) Table of odd ratios, confidence intervals, and Wald tests on the predictor variables. The significant results are indicated in bold. (b) Graphical representation of the results for all the quantitative variables. (c) Graphical representation of the owner‐reported frequencies of birds returned home according to the three environments. Different letters indicate significant differences evidenced in the post hoc tests (including a holm correction). (d) Graphical representation of the owner‐reported frequencies of birds returned home in the six studied breeds. No ped non‐pedigree, BEN, Bengal; BRI, British shorthair; MCO, Maine coon; PER, Persian; RAG, ragdoll; SBI, Birman. Letters indicate significative differences evidenced in the post hoc tests (including a holm correction).

**FIGURE 3:**
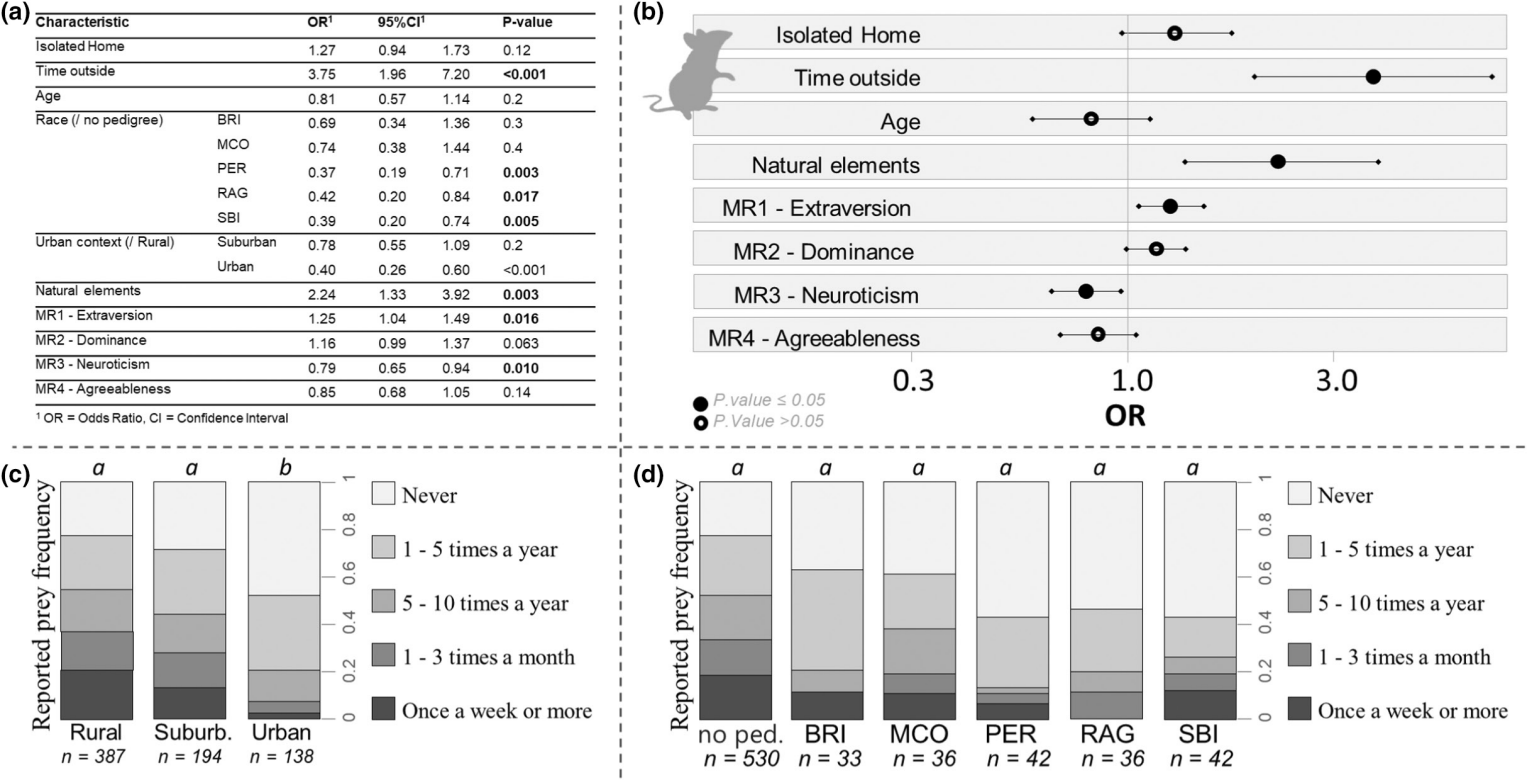
Results of the final CLM2 model regarding the owner‐reported frequencies of mammals returned home (*n* = 719). (a) Table of odd ratios, confidence intervals, and Wald tests on the predictor variables. The significant results are indicated in bold. (b) Graphical representation of the results for all the quantitative variables. (c) Graphical representation of the owner‐reported frequencies of mammals returned home according to the three environments. Different letters indicate significant differences evidenced in the post hoc tests (including a holm correction). (d) Graphical representation of the owner‐reported frequencies of mammals returned home in the six studied breeds. Non‐ped non‐pedigree, BEN, Bengal; BRI, British shorthair; MCO, Maine coon; PER, Persian; RAG, ragdoll; SBI, Birman. Letters indicate significant differences evidenced in the post hoc tests (including a holm correction).

Following the model selection, for the frequency of birds returned home, the final model retained nine variables (cat breed, cat age, abundance of natural elements, urban level, time spent daily by the cat outdoors, and the four personality traits; complete results in Figure 2) and for the frequency of small mammals (mice, field mice, shrew, etc.) returned home, 10 variables (the same previous nine with the addition of the type of housing; complete results in Figure 3). The estimates of the effects of each predictor and the results of Wald's tests are presented in the text below, with the odds ratios and confidence intervals provided in Figures 2 and 3.

Cats with high extraversion had higher bird return rates (estimate = 0.192, SE = 0.095, *z*‐value = 2.032, *p* value = .042) and higher mammal return rates (estimate = 0.220, SE = 0.091, *z*‐value = 2.406, *p* value = .016). Cats with high dominance had higher bird return rates (estimate = 0.194, SE = 0.084, z‐value = 2.313, *p* value = .021) and higher mammal return rates (tendency; estimate = 0.151, SE = 0.081, *z*‐value = 1.858, *p* value = .063). Cats with high neuroticism had lower bird return rates (estimate = −0.253, SE = 0.097, *z*‐value = −2.599, *p* value = .009) and lower mammal return rates (estimate = −0.242, SE = 0.094, *z*‐value = −2.582, *p* value = .010). Cats with high agreeableness had lower bird return rates (estimate = −0.321, SE = 0.114, *z*‐value = −2.829, *p* value = .005).

Cats from an urban environment had lower bird return rates (Rur‐Urb: estimate = 0.977, SE = 0.215, *z*‐value = 4.543, *p* value <.001; Periurb‐Urb‐: estimate = 0.858, SE = 0.229, *z*‐value = 3.750, *p* value <.001) and lower mammal return rates (Rur‐Urb: estimate = 0.924, SE = 0.214, *z*‐value = 4.324, *p* value <.001; Periurb‐Urb: estimate = 0.675, SE = 0.218, *z*‐value = 3.097, *p* value = .004) than those from rural or suburban environments, although no differences were found between rural and suburban environments (Table S1). Owners of cats located in environments rich in natural elements and spending more time outside reported higher bird return rates (Natural elements: estimate = 0.740, SE = 0.303, *z*‐value = 2.442, *p* value = .015, Time outside: estimate = 1.472, SE = 0.335, *z*‐value = 4.390, *p* value <.001) and higher mammal return rates (Natural elements: estimate = 0.807, SE = .273, *z*‐value = 2.956, *p* value = .003, Time outside: estimate = 1.322, SE = 0.330, *z*‐value = 4.006, *p* value <.001). Non‐pedigree cats reported higher bird return rates than Ragdolls (estimate = 1.610, SE = 0.439, *z*‐value = 3.669, *p* value = .004; Figure 2d), although there was no significant difference between breeds for mammal return rates after correcting the p values in post hoc tests (Figure 3d; Table S1).

## 4 DISCUSSION

### 4.1 Personality structure and breed differences

We used a subset of the personality adjectives selected by Litchfield et al. (2017) to assess personality traits in domestic cats. As our exploratory factor analysis revealed only four factors that were very similar to the traits of Litchfield et al., we kept their labels: extraversion (MR1), which reflects a high level of intelligence and perseverance; dominance (MR2), which reflects aggressiveness toward other cats; neuroticism (MR3), which reflects high levels of shyness and fear of other cats; and agreeableness (MR4), which reflects friendliness to people. However, we did not detect the impulsiveness factor of Litchfield et al. (2017). Even when we tried performing exploratory factor analysis with five factors, the additional factor did not correspond to impulsiveness (Table S2). The impulsiveness factor was expected to emerge from the adjectives impulsive, predictable (negative loading), and distractible. However, in our dataset, impulsive was strongly loaded on the dominance factor, whereas predictable and distractible were moderately loaded on the agreeableness factor (Table 1a). Litchfield et al. (2017) analyzed survey data from New Zealand and Australian owners. When the two data sets were examined separately in the initial analysis, the scree plot of both datasets supported retaining only four factors as in the case of our dataset. Furthermore, in this initial separate analysis of the two data sets, the impulsive adjective was strongly and positively loaded on the impulsivity factor in the Australian data set but negatively in the New Zealand data set. It is therefore possible that cat impulsivity is perceived differently in different countries.

Our analyses showed that cats from different breeds tend to have different personality traits, with non‐pedigree and Bengal cats showing a higher tendency toward dominance (MR2) and extraversion (MR1) but a lower tendency toward agreeableness (MR4) than other cats. Bengal and Main Coon cats also demonstrate lower levels of neuroticism than other cats. These findings are in agreement with organizations of cat owners and breeders, which report that cat breeds differ not only in morphological traits but also in behavior (Salonen et al., 2019). For example, Bengal cats are described as intelligent and active, while Birman cats are described as affectionate and gentle. Recently, Salonen et al. (2019) used surveys to examine behavioral differences between breeds in a sample of over 5700 Finnish cats from 19 different breeds and detected differences between breeds in all traits relating to social and non‐social behavior. Even though our results are less detailed than those of Salonen et al., because we examined fewer breeds with a smaller number of behavioral dimensions, they nevertheless seem to be in good agreement. For example, we observed that the Ragdoll, Persian, and British Shorthair cats generally seem to be more agreeable, less dominant, and less extraverted than other breeds of cats (Figure S1). These breeds were previously shown to be closely related genetically (Lipinski et al., 2008) and to have low levels of activity and aggression (Salonen et al., 2019).

### 4.2 Factors influencing owner‐reported frequency of prey brought home

It was previously shown that domestic cats have highly variable predation rates: some cats frequently bring prey home, while a significant proportion rarely does so (Baker et al., 2008; Kauhala et al., 2015; Thomas et al., 2012; Tschanz et al., 2011). Given the high local ecological impact of pet cat predation, understanding the causes of this variation could potentially help identify ways of mitigating this impact. Variations in hunting behavior among pet cats are related to three main factors: (1) the availability of prey in the environment (e.g., Barratt, 1997; Bonnaud et al., 2009); (2) the practices of owners, who can influence the cats' access to prey by regulating their outdoor access and using deterrents and can affect the cats' motivation to hunt by providing a suitable diet or enriching the environment (Cecchetti et al., 2021b); and (3) the intrinsic characteristics of the cats themselves, which modulate their reactions to the previous factors. In this study, we focused on the effect of cat characteristics (personality traits in addition to sex, age, and breed) and included several factors related to the cats' environment (see ‘Section 2’), which were expected to play an important role.

Regarding the relationship between the individual characteristics and the reported predation rate of cats, we observed a significant effect of breed in addition to the four personality traits studied here. By contrast, age and sex did not seem to play a significant role, as also observed by Cordonnier et al. (2022). Note, however, that we excluded cats younger than 1 year from the analyses, while their sexed and desexed status was not recorded. The main finding of this study is that cat personality has a major influence on the owner‐reported frequency of birds and small mammals brought home, which, to our knowledge, has not previously been observed. For both birds and small mammals, cats with high levels of extraversion (here, intelligence and perseverance) had significantly higher frequencies of prey brought home, whereas cats with high levels of neuroticism (shyness and fear of other cats) had significantly lower frequencies. Thus, our findings clearly confirm the hypothesis of Cecchetti et al. (2021a) that cats with low levels of neuroticism or high levels of extraversion hunt wild prey more frequently. Additionally, we observed that cats with low levels of agreeableness (here, friendliness to people) and high levels of dominance had higher frequencies of bringing home birds but not small mammals.

The personality of cats can potentially influence their predation activity at several different levels. First, it can modulate the time that cats choose to spend outside. For example, cats with high neuroticism could be more fearful of going outdoors than other cats, or friendly cats with high agreeableness could be more motivated to stay inside with their owners. Lowe and Bradshaw (2001) thus showed that “staying indoors” is an important element of the behavioral styles recognizable in young domestic cats. Second, personality can also influence the time that the owners allow to their cats to spend outdoors (Tan et al., 2021). In their large international study, Foreman‐Worsley et al. (2021) showed that “Some owners felt their cat's temperament made them unsuitable to go outdoors.” For example, owners of timid cats tended to keep them indoors more to avoid their cat being “bullied.” Furthermore, Foreman‐Worsley et al. (2021) observed that the large majority of owners who allowed their cat to go outside did so because they believed that their cat wanted outdoor access, again suggesting a relationship between cat personality and the amount of outdoor time allowed by the owners. Third, during the time spent outdoors, personality can also influence cat motivation to hunt. For example, fearful cats with high neuroticism could be less likely to venture far from home and thus locate prey. Although not demonstrated in domestic cats to our knowledge, the effects of personality on space use have been shown in several species (e.g., Marmet et al., 2012; Schirmer et al., 2019; Wauters et al., 2021). Finally, cats with personality traits such as high levels of intelligence and perseverance could be more successful hunters. Our data set does not allow us to disentangle these four possibilities. However, a survey with a larger sample, particularly a larger sample of free‐ranging cats, would make it possible to determine whether the observed effect of personality traits on the frequency of prey brought home is primarily mediated by the time spent outdoors or by a greater motivation or hunting efficiency once outdoors.

Regarding the cats' environment, we found that rural or suburban settings as opposed to an urban environment and a high abundance of vegetation around the home were associated with higher frequencies of prey brought home as reported by the owners. As expected, we also found that cats who spent a greater amount of time outdoors had higher reported frequencies of prey brought home (though cats without outdoor access were excluded from this analysis). Because pet cats usually remain close to their home (~100 m radius in average; Kays et al., 2020) and are opportunistic hunters, their predation should reflect the fauna found in immediate proximity to their home (Barratt, 1997; Castañeda et al., 2019, 2020). Several studies on free‐ranging pet cats found significant differences between rural and urban areas in terms of the amount and composition of prey brought home, probably reflecting differences in local prey availability induced by differences in land use (Kauhala et al., 2015; Krauze‐Gryz et al., 2017; Piontek et al., 2021). In our study, predation analysis was conducted on cats with outdoor access ranging from <1 h per day to free‐ranging cats. This has the advantage of being more representative of the pet cat population as a whole because not all pet cats are free ranging. However, because owners living in urban settings are much more likely to limit their cat's time spent outdoors, often due to their fear of road traffic accidents (Foreman‐Worsley et al., 2021), this means that the effects of urban and rural environments as well as the time spent outdoors are difficult to separate in our data set.

### 4.3 Limitations

In this study, we used online convenience sampling to survey cat owners about their animals' personality traits as well as the frequency of prey brought home. This methodology allowed us to gather a large sample, although it also has several limitations. First, we contacted respondents through social media by disseminating the questionnaire in user groups dedicated to cats. However, the sociodemographic characteristics of these social media users probably differ from those of the general population, for example, in terms of age and education level (Mellon & Prosser, 2017). Furthermore, it is likely that the participants in the cat‐dedicated groups present differences in terms of their relationship to their cat (high interest in particular) compared with cat owners who do not frequent such groups. It is therefore likely that the respondents do not constitute a representative sample of French cat owners. In addition, we estimated cat predatory activity using a semi‐quantitative measure of how often they bring prey home, as observed by their owners. Although cat predation rates are frequently estimated by the prey brought home method (e.g., Krauze‐Gryz et al., 2017; Lepczyk et al., 2004; Tschanz et al., 2011; Woods et al., 2003), this approach has limitations. In particular, cats only bring home a fraction of the prey that they capture. For example, Loyd et al. (2013) monitored free‐ranging pet cats in a suburban area of the southeastern USA for 1 year using KittyCam video cameras and showed that only 23% of the prey captured by cats were brought home, while 49% were left behind and 28% consumed. Furthermore, the proportion of prey brought home varies between prey groups, especially in terms of how palatable they are (Krauze‐Gryz et al., 2012). Similarly, Seymour et al. (2020) recently showed that in Cape Town, South Africa, 82% of pet cats' prey was not returned home, again with very different proportions of prey returned by taxa. For these reasons, owner surveys of prey brought home by their cat markedly underestimate the amount of prey captured depending on the type of prey. However, in this study, we were interested in determining which personality factors contributed to the variations in predation rates between cats as opposed to the absolute amount of prey captured by the animals. These limitations are therefore not supposed to impact the results of the present research.

## 5 CONCLUSION

The major influence of cat personality on the frequency of birds and small mammals brought home could potentially help mitigate predation by domestic cats. Pet cat predation rates are strongly associated with the amount of time spent outdoors, although other factors are also important (Cecchetti, Crowley, Goodwin, & McDonald, 2021; Cecchetti, Crowley, & McDonald, 2021). While the situation varies from country to country, several authors have shown that owners' decisions about whether or not to allow their cats to go outside are rarely motivated by a consideration of their cats' impact on wildlife (Crowley et al., 2019; Foreman‐Worsley et al., 2021). Foreman‐Worsley et al. (2021) showed that in several countries, the main reason for owners keeping their cats indoors was to protect them from traffic, especially in urban environments, whereas the main reasons for allowing them to go outside are the perceived mental health benefit or because the cats wanted to go outside. Crowley et al. (2019) showed that British cat owners who wanted to manage their cats' predation were often concerned that it would compromise their pets' welfare. The same authors (Crowley et al., 2020b) also recently observed that the majority of surveyed owners “valued outdoor access for cats and opposed confinement to prevent hunting.” Taking into account the personality of the cats having an outdoor access, for instance by promoting the adoption of cats (or breeds) that are by temperament less likely to hunt (cats with low extraversion and dominance, but high neuroticism and high agreeableness), could therefore potentially allow owners to reduce the impact of their cats on wildlife in places where there are strong biodiversity preservation issues.

## AUTHOR CONTRIBUTIONS

**Marion Cordonnier:** Conceptualization (equal); data curation (equal); formal analysis (equal); methodology (equal); visualization (equal); writing – original draft (equal); writing – review and editing (equal). **Amira Perrot:** Conceptualization (equal); data curation (equal); formal analysis (equal); methodology (equal); validation (equal); visualization (equal); writing – review and editing (equal). **Nicolas Ferry:** Methodology (equal); validation (equal); writing – review and editing (equal). **Elsa Bonnaud:** Methodology (equal); validation (equal); writing – review and editing (equal). **Emmanuelle Baudry:** Conceptualization (equal); methodology (equal); supervision (equal); writing – original draft (equal); writing – review and editing (equal).

### OPEN RESEARCH BADGES

This article has earned an Open Data badge for making publicly available the digitally‐shareable data necessary to reproduce the reported results. The data is available at https://data.mendeley.com/datasets/ht5p5pg7b7/1.

## Supporting information


Appendix S1

## Data Availability

The data that support the findings of this study are openly available in Mendeley repository (DOI: https://doi.org/10.17632/ht5p5pg7b7.1; https://data.mendeley.com/datasets/ht5p5pg7b7)"
