# Supplementary material for: Pet cat personality linked to owner‐reported predation frequency
Source: Ecol Evol. 2023 Jan 24;13(1):e9651. doi: 10.1002/ece3.9651 (PMC9873523; doi:10.1002/ece3.9651)
Supplement: Supplementary file 1 — Appendix S1 [file ECE3-13-e9651-s001.docx]

**Appendix 1: Survey**

This English version of the questionnaire is a translation of the French version that was used in the study.

**STUDY OF PERSONALITY AND PREDATION IN DOMESTIC CATS**

Welcome to the Cat Personality survey and thank you for taking part!

This survey will help us to understand more about the personality and the predation of domestic cats. We will ask you questions about the characteristics of your cat, its behavior and its environment. The survey will take approximately 5 minutes to complete. Response to this survey is voluntary and your responses will be kept confidential.

By completing this questionnaire, you are indicating your consent to participate in the Cat Personality study. Individual’s responses will be kept confidential by the researchers (Dr Emmanuelle Baudry, University Paris Saclay, France) and will not be identified in the reporting of the research.

If you would like to contact the researcher, Dr Emmanuelle Baudry, or if you have any question please send an email to Emmanuelle.baudry@universite-paris-saclay.fr

Warning: If you have several cats, please focus on one.

**Characteristics of your cat**

*The following questions will help us to determine the key characteristics of your cat.*

**Sex** Female/ Male/ Unknown

**Age** Less than 1 year/ 1-2 years/ 2-10 years/ More than 10 years/ Unknown

**Breed** Bengal/ Birman/ British Shorthair/ Chartreux / European/ Maine coon / Persian/ Ragdoll/ Savannah / Sphynx/ Siamese/ Turkish angora / No breed / Other / Unknown

**How many pet cats do you have?** 1/ 2/ 3/ 4/ 5/ More than 5

**Living conditions of your cat**

*The following questions will help us to understand the environment of your cat.*

**What is your country of residence?**

**You live in** Apartment without balcony / Apartment with balcony or terrace/ House in a subdivision / Individual house

**Would you say that you live in an area that is** Urban / Suburb / Rural

**How much time does your cat spend each day outdoors?** None / Limited (less than one hour)/ Moderate (1 to 5 hours)/ Long (more than 5 hours)/ All the time (come back just to eat)

**How much time do you spend each day with you cat (observation, game, petting, care, ...)?**

None / Limited (less than one hour)/ Moderate (1 to 5 hours)/ Long (more than 5 hours)

**Personality of your cat**

*To describe the personality of your cat, here is a list of adjectives.*

|  | Strongly disagree | Disagree | Neither agree nor disagree | Agree | Strongly agree |
| --- | --- | --- | --- | --- | --- |
| Shy |  |  |  |  |  |
| Calm |  |  |  |  |  |
| Fearful of other cats |  |  |  |  |  |
| Smart |  |  |  |  |  |
| Vigilant |  |  |  |  |  |
| Persevering |  |  |  |  |  |
| Affectionate |  |  |  |  |  |
| Friendly to people |  |  |  |  |  |
| Solitary |  |  |  |  |  |
| Bullying |  |  |  |  |  |
| Dominant |  |  |  |  |  |
| Aggressive to other cats |  |  |  |  |  |
| Impulsive |  |  |  |  |  |
| Predictable |  |  |  |  |  |
| Distractible |  |  |  |  |  |

**Predatory behavior of you cat**

*The following questions will help us to estimate the predation of your cat on birds and small mammals.*

**Around your place of residence, would you say that abundance of natural areas (tree, bushes, grass ...) is** Low / Moderate/ High/ I don’t know

**At what frequency does your cat capture birds?** Never/ Rarely (1 to 5 times a year)/ Sometimes (5 to 10 times a year)/ Often (1 to 3 times a month)/ Very often (once a week or more)

**At what frequency does your cat capture small mammals (mice, field mice, shrew)?** Never/ Rarely (1 to 5 times a year)/ Sometimes (5 to 10 times a year)/ Often (1 to 3 times a month)/ Very often (once a week or more)

**Would you like to add something else about your cat?** (open ended)

**Thank you very much for participating in our survey!**

This French version of the questionnaire is the one that was used in the study**.**

**Etude de la personnalité et de la prédation chez le chat**

Bienvenue dans le questionnaire et merci de votre participation.

Ce questionnaire vise à étudier, dans le cadre d'une étude scientifique, la personnalité et la prédation des chats domestiques. Il faut approximativement 5 minutes pour le compléter.

En remplissant ce questionnaire, vous consentez à participer à l’étude portant sur la personnalité des chats et leur prédation. Chacune de vos réponses sont anonymes et gardées confidentielles par le chercheur (Dr Emmanuelle Baudry, Université Paris-Saclay). Si vous souhaitez contacter le chercheur, Dr Emmanuelle Baudry, ou si vous avez des questions, vous pouvez envoyer un email à Emmanuelle.Baudry@universite-paris-saclay.fr

Attention : Si vous possédez plusieurs chats, merci de vous concentrer sur un seul, celui de votre choix. Si vous le souhaitez, vous pouvez refaire ce questionnaire ensuite pour un autre de vos chats.

**Caractéristiques de votre chat**

*Les questions suivantes nous permettent d'obtenir les caractéristiques principales de votre chat.*

**Sexe** Femelle / Mâle/ Ne sait pas

**Age** Moins de 1 an/1-2 ans/2-10 ans/Plus de 10 ans

**Race** Angora turc/ Bengale/ Birman/ British Shorthair/ Chartreux / Européen/ Maine coon / Persan/ Ragdoll/ Savannah / Sphynx/ Siamois/ Pas de race / Autre race / Ne sait pas

**Combien de chats possédez-vous ?** 1/ 2/ 3/ 4/ 5/ Plus de 5

**Lieu de vie de votre chat**

*Les questions suivantes nous permettent de comprendre le lieu et les habitudes de vie de votre chat.*

**Dans quel type de logement habitez-vous ?** Appartement sans balcon/ Appartement avec balcon ou terrasse/ Maison en lotissement/ Maison isolée

**Diriez-vous que vous habitez plutôt en zone** Urbaine/ Périurbaine/ Rurale

**Quelle est la durée que votre chat passe à l'extérieur par jour ?** Aucune/ Réduite (moins de 1 heure) / Modérée (1 à 5 heures) / Elevée (plus de 5 heures) / Tout le temps (ne revient que pour manger)

**Combien de temps passez-vous avec votre chat par jour (observation, jeu, caresse, soin, ...) ?** Aucun/ Réduit (moins de 1 heure) / Modéré (1 à 5 heures) / Elevée (plus de 5 heures)

**Personnalité de votre chat**

*Pour décrire la personnalité de votre chat, voici une liste d'adjectifs.*

|  | *Pas du tout d’accord* | *Plutôt pas d’accord* | *Ni d’accord, ni pas d’accord* | *Plutôt d’accord* | *Tout à fait d’accord* |
| --- | --- | --- | --- | --- | --- |
| Timide |  |  |  |  |  |
| Calme |  |  |  |  |  |
| Effrayé par les autres chats |  |  |  |  |  |
| Intelligent |  |  |  |  |  |
| Vigilant |  |  |  |  |  |
| Persévérant |  |  |  |  |  |
| Affectueux |  |  |  |  |  |
| Amical avec les gens |  |  |  |  |  |
| Solitaire |  |  |  |  |  |
| Brutal |  |  |  |  |  |
| Dominant |  |  |  |  |  |
| Aggressif envers les autres chats |  |  |  |  |  |
| Impulsif |  |  |  |  |  |
| Prévisible |  |  |  |  |  |
| Facilement distrait |  |  |  |  |  |

**Prédation de votre chat**

*Les questions suivantes nous permettent de quantifier la prédation de votre chat sur les oiseaux et/ou sur les petits mammifères tels que les rongeurs.*

**Concernant votre extérieur, vous diriez que l'abondance d'éléments naturels (arbres, buissons, herbe...) est plutôt** Faible/ Modérée/ Elevée/ Ne sait pas

**A quelle fréquence votre chat capture-t-il des oiseaux ?** Jamais/ Très peu (1 à 5 fois par an) / Peu (5 à 10 fois par an) / Modérée (1 à 3 fois par mois) / Elevée (1 fois par semaine ou plus)

**A quelle fréquence votre chat capture-t-il des petits mammifères (mulot, musaraigne, souris ...) ?**

Jamais/ Très peu (1 à 5 fois par an) / Peu (5 à 10 fois par an) / Modérée (1 à 3 fois par mois) / Elevée (1 fois par semaine ou plus)

**Vous pouvez si vous le souhaitez ajouter une particularité de votre chat qui n’a pas été traitée dans le questionnaire.** (question ouverte)

**Merci beaucoup pour votre participation!**

**Appendix Figure 1: Discriminant analysis of the relationship between breed and personality.**


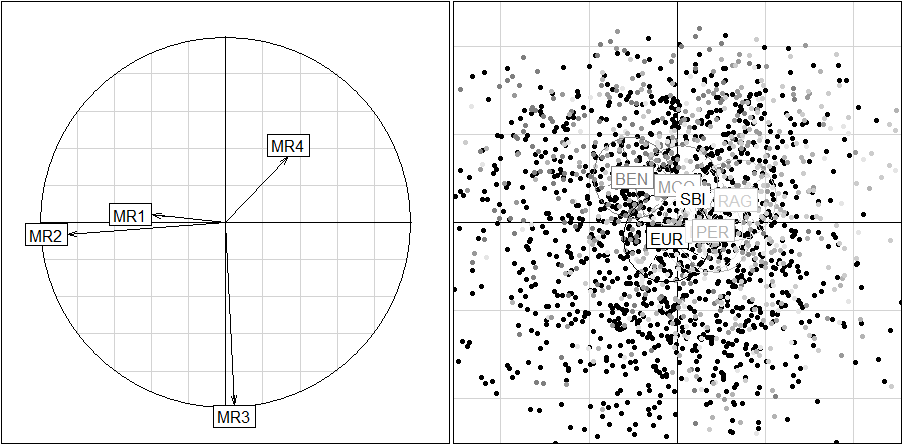


MR1: extraversion; MR2 dominance; MR3 neuroticism; MR4 agreableness

EUR non-pedigree, BEN Bengal, BRI British Shorthair, MCO Maine coon, PER Persian, RAG Ragdoll, SBI Birman

Left: variable plot; right individuals plot.

**Appendix Table 1: frequency of prey returned home, post-hoc tests for the categorical variables**

Birds

contrast estimate SE df z.ratio p.value

EURvsBRI 0.2623 0.361 Inf 0.727 1.0000

EURvsMCO 0.5133 0.357 Inf 1.439 1.0000

EURvsPER 0.7553 0.357 Inf 2.118 0.4105

EURvsRAG 1.6097 0.439 Inf 3.669 **0.0037**

EURvsSBI 0.8396 0.349 Inf 2.408 0.2086

BRIvsMCO 0.2510 0.486 Inf 0.516 1.0000

BRIvsPER 0.4929 0.485 Inf 1.017 1.0000

BRIvsRAG 1.3473 0.547 Inf 2.462 0.1932

BRIvsSBI 0.5773 0.475 Inf 1.216 1.0000

MCOvsPER 0.2419 0.482 Inf 0.501 1.0000

MCOvsRAG 1.0963 0.546 Inf 2.008 0.4914

MCOvsSBI 0.3263 0.474 Inf 0.689 1.0000

PERvsRAG 0.8544 0.543 Inf 1.573 1.0000

PERvsSBI 0.0844 0.473 Inf 0.178 1.0000

RAGvsSBI -0.7700 0.535 Inf -1.439 1.0000

RuralvsSuburban 0.1190 0.170 Inf 0.700 0.4840

RuralvsUrban 0.9770 0.215 Inf 4.543 **<.0001**

SuburbanvsUrban 0.8580 0.229 Inf 3.750 **0.0004**

Mammals

contrast estimate SE df z.ratio p.value

EURvsBRI 0.3737 0.351 Inf 1.063 1.0000

EURvsMCO 0.2992 0.340 Inf 0.880 1.0000

EURvsPER 0.9987 0.337 Inf 2.963 **0.0456**

EURvsRAG 0.8701 0.363 Inf 2.396 0.2154

EURvsSBI 0.9510 0.339 Inf 2.804 0.0707

BRIvsMCO -0.0745 0.469 Inf -0.159 1.0000

BRIvsPER 0.6250 0.465 Inf 1.345 1.0000

BRIvsRAG 0.4964 0.482 Inf 1.030 1.0000

BRIvsSBI 0.5773 0.464 Inf 1.245 1.0000

MCOvsPER 0.6995 0.455 Inf 1.536 1.0000

MCOvsRAG 0.5709 0.477 Inf 1.198 1.0000

MCOvsSBI 0.6518 0.455 Inf 1.432 1.0000

PERvsRAG -0.1286 0.469 Inf -0.274 1.0000

PERvsSBI -0.0478 0.451 Inf -0.106 1.0000

RAGvsSBI 0.0808 0.468 Inf 0.173 1.0000

RuralvsSuburban 0.2490 0.173 Inf 1.434 0.1515

RuralvsUrban 0.9240 0.214 Inf 4.324 **<.0001**

SuburbanvsUrban 0.6750 0.218 Inf 3.097 **0.0039**

EUR non-pedigree, BEN Bengal, BRI British Shorthair, MCO Maine coon, PER Persian, RAG Ragdoll, SBI Birman

**Appendix Table 2: exploratory factor analysis with 5 factors**

MR1 MR2 MR3 MR5 MR4 h2 u2 com

Timide 0.04 -0.05 **0.75** 0.10 0.06 0.58 0.42 1.1

Calme 0.04 -0.03 0.09 **0.66** 0.00 0.49 0.51 1.0

Effrayé 0.01 0.05 **0.49** 0.16 0.19 0.30 0.70 1.6

Intelligent **0.74** -0.02 -0.08 0.06 0.01 0.58 0.42 1.0

Vigilant **0.74** 0.02 0.23 0.02 -0.07 0.58 0.42 1.2

Perséverant **0.71** 0.04 -0.09 -0.12 0.02 0.51 0.49 1.1

Affectueux 0.38 -0.09 -0.20 0.25 0.30 0.47 0.53 3.5

Amical 0.06 -0.01 **-0.58** 0.22 0.24 0.49 0.51 1.7

Solitaire 0.05 0.31 0.35 0.19 -0.16 0.29 0.71 3.0

Brutal 0.02 **0.45** 0.01 -0.33 0.08 0.40 0.60 1.9

Dominant 0.07 **0.70** -0.20 -0.03 -0.09 0.52 0.48 1.2

Agressif -0.05 **0.73** 0.10 0.14 0.00 0.51 0.49 1.1

Impulsif 0.04 **0.56** 0.06 -0.27 0.22 0.55 0.45 1.8

Prévisible 0.06 0.09 -0.02 0.19 0.33 0.18 0.82 1.9

Distrait -0.06 0.02 0.07 -0.09 **0.57** 0.31 0.69 1.1

Salient factor loadings (above |0.40|) are shown in bold. h2 measures communalities, the shared variance with the other items, whereas u2 measures uniqueness, the variance not explained by the other items
